# Supplementary material for: EZH2 specifically regulates ISL1 during embryonic urinary tract formation
Source: Sci Rep. 2024 Oct 2;14:22909. doi: 10.1038/s41598-024-74303-w (PMC11447050; doi:10.1038/s41598-024-74303-w)
Supplement: Supplementary file 1 — Supplementary Material 1 [file 41598_2024_74303_MOESM1_ESM.pdf]

Supplementary information

EZH2 specifically regulates *ISL1* during embryonic urinary tract formation

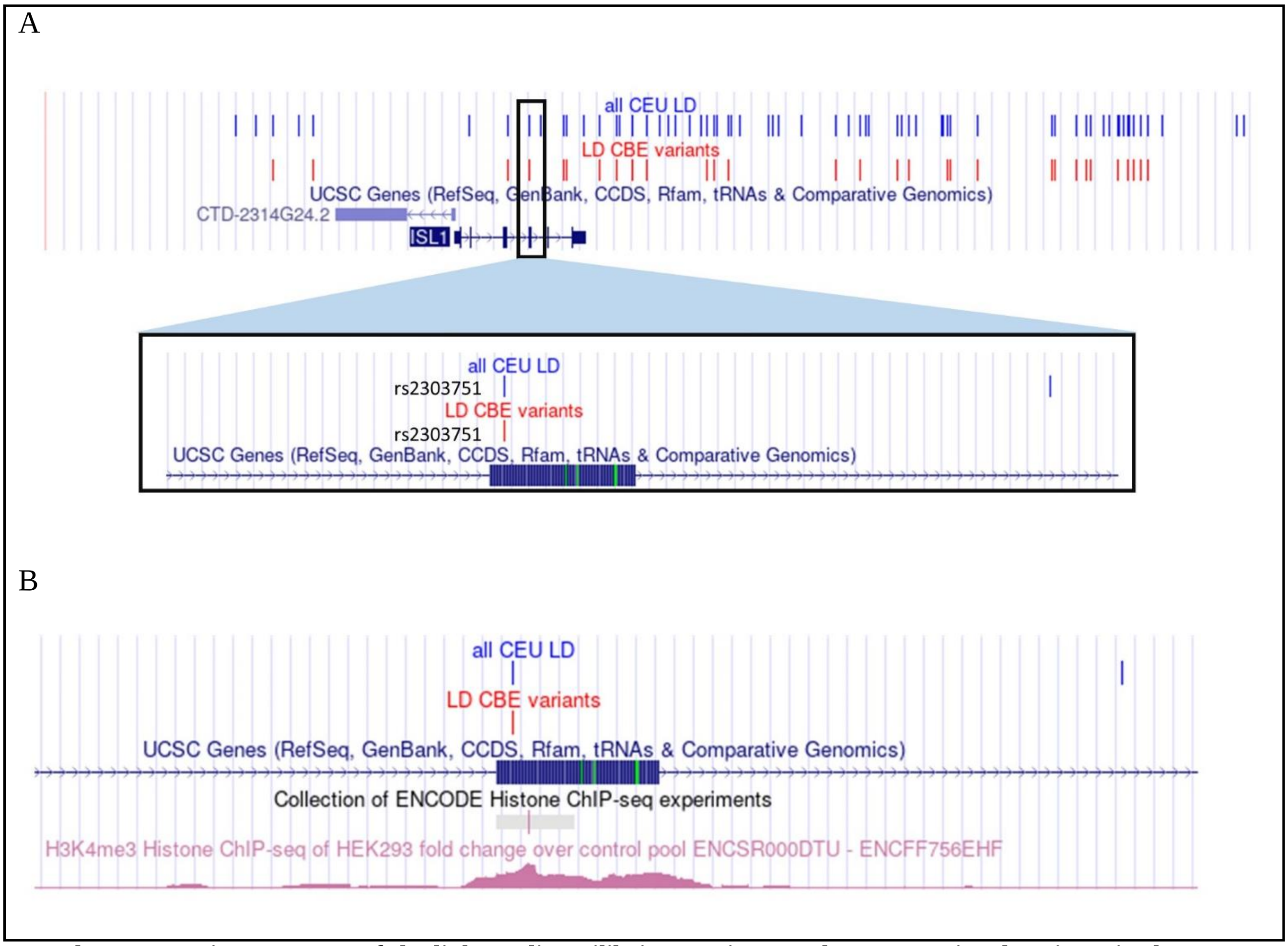

Supplementary Figure 1. Map of the linkage disequilibrium variants and CBE associated variants in the ISL1 candidate region. A) Top panel: All CEU LD (in blue) shows all the variant in linkage disequilibrium (LD) with the top CBE associated rs6874700 variant; LD CBE variants (in red) shows CBE GWAS variants that are in common with the ones in LD. Bottom panel: zoom in the region where the only regulatory variant rs2303751 reseeds. B) H3K4me3 marker in the rs2303751 region displays a significant peak.

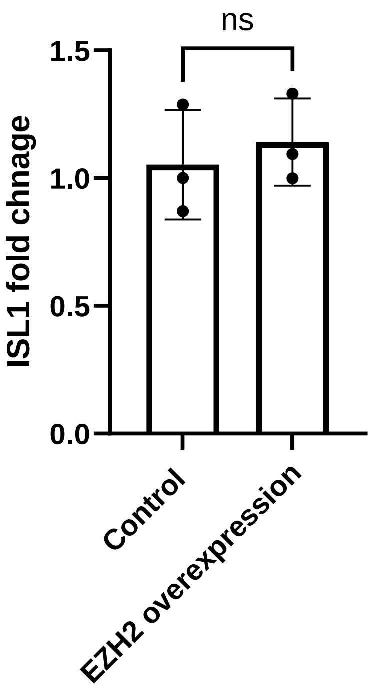

Supplementary Figure 2. *ISL1* expression with EZH2 overexpression. qPCR of ISL1 with control and EZH2 overexpression in HEK293 cells shows no difference in ISL1 gene regulation

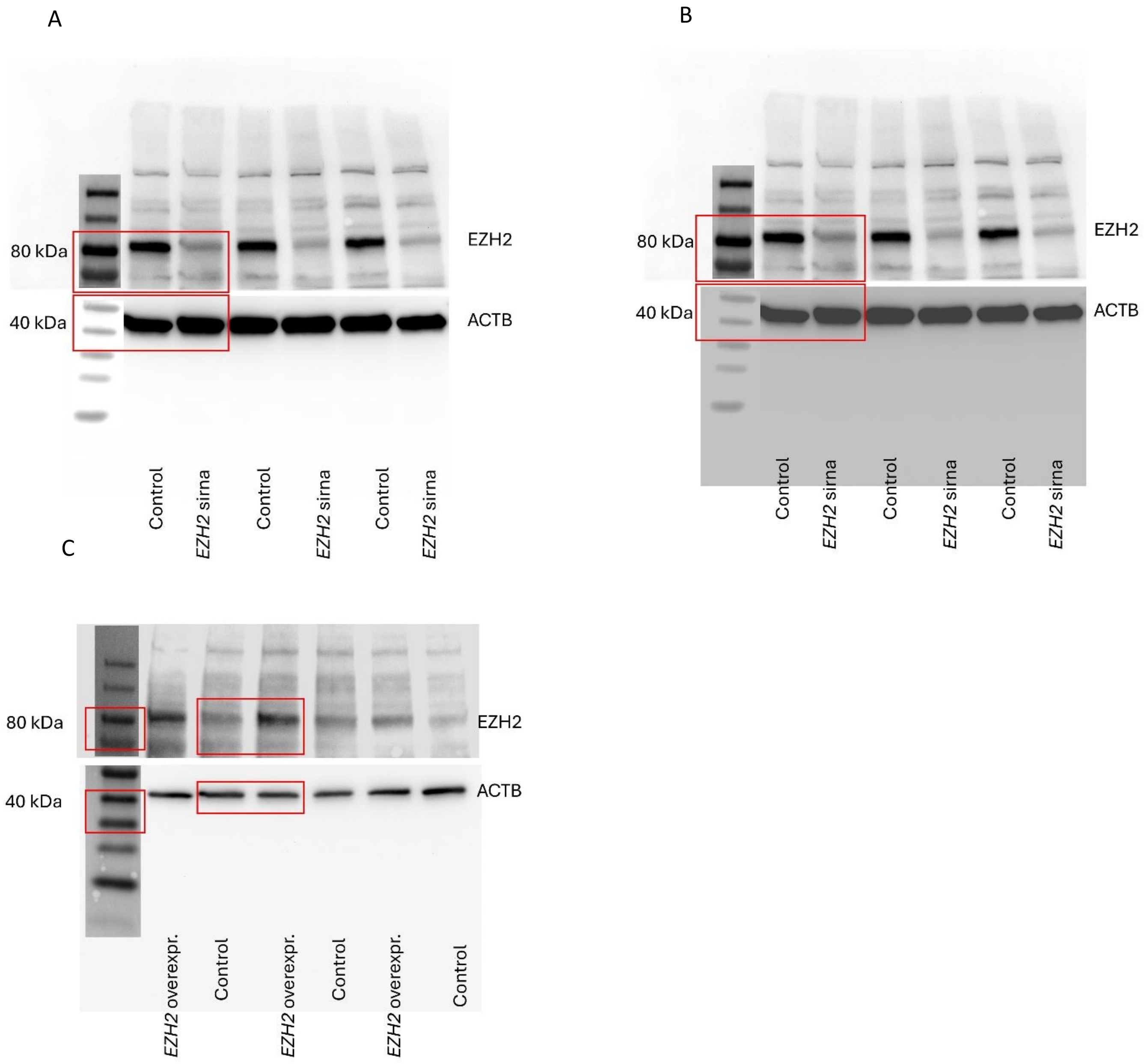

Supplementary Figure 3. Full western blot membranes of *EZH2* silencing and overexpression as triplicate. A and B shows *EZH2* silencing with different contrast on beta-acting protein. Marker on the beta actin (capure in brightfield) partially overlay the blots due to software brightfield-capturing area. C shows *EZH2* overexpression. All blots are showed with low contrast. Red boxes indicate the cropped regions of the blots presented in the main manuscript.

Oligonucleotides

Name	Sequence (5' to 3')	Usage
zf_EZH2_Talen_F	AAATCGGAGAAGGGTCCTG	ezh2 larvae genotyping
zf_EZH2_Talen_R	ACACACATGCAACTGGACTC	ezh2 larvae genotyping
isl1_Fragment2_XhoI_FLP_F	catcatACGCGTGACTTTGAGACCTGCTTCCCTTG	Luciferase cloning
sl1_Fragment2_MluI_FLP_R	catcatCTCGAGTAACTTCACCAGGAGGCCTGC	Luciferase cloning
isl1_Fragment2_XhoI_F	catcatACGCGTGACTTTGAGACCTGCTTCCCTTG	Luciferase cloning
isl1_Fragment2_MluI_R	catcatCTCGAGTAACTTCACCAGGAGGCCTGC	Luciferase cloning
P8_Fragment1_XhoI_F	tcatACGCGTAGGTGTTGGCCTGACCCTAGGG	Luciferase cloning
P9_Fragment1_MluI_R	tcatCTCGAGGTTTGCGGCGTAGCAGGTCCG	Luciferase cloning
P8 FLP_Fragment1_MluI_F	tcatCTCGAGTAGGTGTTGGCCTGACCCTAGGG	Luciferase cloning
P9 FLP_Fragment1_XhoI_R	tcatACGCGTGGTTTGCGGCGTAGCAGGTCCGC	Luciferase cloning
P17_Fragment3_XhoI_F	tcatACGCGTTGCCCCTCATCCTTACCCCC	Luciferase cloning
P18_Fragment3_MluI_R	tcatCTCGAGTCGTGTCTCTCTGGACTGGCAG	Luciferase cloning
P17 FLP_Fragment3_MluI_F	tcatCTCGAGTGCCCCTCATCCTTACCCCC	Luciferase cloning
P18 FLP_Fragment3_XhoI_R	tcatACGCGTGTCGTGTCTCTCTGGACTGGCAG	Luciferase cloning
ACTB_Hum_1F	CTTCCTTCCTGGGCATGGAG	qPCR
ACTB_Hum_1R	AGCACTGTGTTGGCGTACAG	qPCR
ISL1 qpcr F2	CAGCAACTGGTCAATTTTTCAG	qPCR
ISL1 qpcr R2	CTCAATAGGACTGGCTACCATG	qPCR
isl1_zf_qPCR_F	CGTGTTTGAAATGTGCAG	qPCR
isl1_zf_qPCR_R	CCGTATAACCTGATGTAGTC	qPCR
zf actb1 qpcr for.	GACACAGATCATGTTCGA	qPCR
zf actb1 qpcr rev.	GCGTAACCCTCATAGATG	qPCR
ISL1-DT_F	GTGTGCTGACCCAAGTGGTG	qPCR
ISL1-DT_R	TGTTTCACTCTCCGGACTGC	qPCR
ar F	CACAGGCTACCTGGTCCT	ChIP qPCR
ar R	TCTGGGACGCAACCTCT	ChIP qPCR
ACTB_ChIP_F	TCTGAACAGACTCCCCATCC	ChIP qPCR
ACTB_ChIP_R	ACCATGTCACACTGGGGAAG	ChIP qPCR
ChIP -1,6 F	AGCATCGGCTTCAGCAAGAAC	ChIP qPCR
ChIP -1,6 R	ATTCGTCCCCAGGGATGAGC	ChIP qPCR
ChIP -1,2 F	TACACCACTTGTGTACACGTG	ChIP qPCR
ChIP -1,2 R	AATTAGGCAGCAGGAGAATACC	ChIP qPCR
ChIP -0,9 F	CCATAGGTGTTGGCCTGACC	ChIP qPCR
ChIP -0,9 R	TCCCGGCCAACCTTGCTGTG	ChIP qPCR
ChIP -0,5 F	GAGCAGGGATTGGAGATATGGC	ChIP qPCR
ChIP -0,5 R	CGAGAACTCTGCCAGAACGC	ChIP qPCR
ChIP +0 F	TGCTGTGAACAGGGGGACAG	ChIP qPCR
ChIP +0 R	CTTTGCTGAGTAATCCCGGCC	ChIP qPCR
ChIP +0,3 F	GCCTCCAGCCCAGCGCTCAC	ChIP qPCR
ChIP +0,3 R	TTCTCCGGCTGCTTGTGGACG	ChIP qPCR
ChIP +0,6 F	AGCAGCAGCAGCCCAATGAC	ChIP qPCR
ChIP +0,6 R	CCTGCGTACCAGGAACGCAC	ChIP qPCR
ChIP +0,9 F	GAACCGGAGAAACGCCGTCC	ChIP qPCR

ChIP +0,9 R	TCCCTCTTCTTGTGTACGTGAG	ChIP qPCR
ChIP +1,2 F	AGATCACCCTCTGCTCCAGG	ChIP qPCR
ChIP +1,2 R	CTTCACCAGGAGGCCTGCAG	ChIP qPCR
ChIP +1,5 F	GTTACAGTATCCCTTGCTGGG	ChIP qPCR
ChIP +1,5 R	TAGAGCTCATGTCCCTTTTGC	ChIP qPCR
ChIP +1,7 F	TCTATCCTGAAACCTTGTTCCC	ChIP qPCR
ChIP +1,7 R	AGAAAGCAAATGCCCTCCAG	ChIP qPCR
pGL3_seq_F	CTAGCAAAATAGGCTGTCCC	sequencing
pGL3_seq_R	CTTTATGTTTTTGGCGTCTTCCA	sequencing
